# Brigade Surgeons

**Published:** 1861-09

**Authors:** 


BRIGADE SURGEONS.—The following gentlemen have been appointed Brigade Surgeons in the Volunteer Service of the United States, their commissions dating from the 3d of August:—

Maine: Daniel McRuer. Massachusetts: George H. Lyman, Henry Bryant, Luther V. Bell, Peter Pineo, O. Martin. Connecticut: P. W. Ellsworth. New York: F. H. Hamilton, Henry S. Hewitt, John A. Lydell, John C. Dalton, Jr., George Suckley, A. H. Hoff, W. H. Church, Rufus H. Gilbert, Charles McMillan, T. Rush Spencer. New Jersey: J. E. Quidor. Pennsylvania: J. H. Brinton, S. W. Gross, A. B. Campbell, N. R. Derby, A. E. Stocker, J. Owen, James King. District of Columbia: J. G. F. Holston, William E. Walters. Ohio: Charles O'Leary, J. D. Robinson, William Glendennin, George G. Shumard. Indiana: J. S. Bobbs, William B. Stewarts, W. C. Thompson. Illinois: David Prince, Joseph W. Freer, J. V. Z. Blaney, Thomas Sim, J. H. Banch, S. R. Haven.

